# Mesenteric Panniculitis Presenting as Fever of Unknown Etiology in a Patient with History of Abdominal Surgery

**DOI:** 10.1155/2018/5658039

**Published:** 2018-01-29

**Authors:** Christopher P. Irwin, Joo B. Lee, Andrew Kim, Ijagha Eme, Christina Schofield, George Mount

**Affiliations:** ^1^Internal Medicine, Madigan Army Medical Center, Tacoma, WA, USA; ^2^Infectious Disease, Madigan Army Medical Center, Tacoma, WA, USA; ^3^Rheumatology, Madigan Army Medical Center, Tacoma, WA, USA

## Abstract

Mesenteric panniculitis is characterized by nonspecific fibrous inflammation of the small bowel mesentery, appendix, and mesoappendix. Clinical course is usually benign and outcome is favorable. We report a case of mesenteric panniculitis presenting as fever of unknown etiology in a patient with history of abdominal surgery.

## 1. Introduction

Mesenteric panniculitis is inflammation of the mesentery, particularly involving the small bowel, appendix, and mesoappendix. Consistent with its representation of a spectrum of disease processes characterized by degeneration, inflammation, and fibrosis of adipose tissue, mesenteric panniculitis has been noted in literature under numerous descriptions. First described as retractile mesenteritis, mesenteric panniculitis is also known as Pfeiffer-Weber-Christian disease, retractile mesenteritis, mesenteric lipodystrophy, and sclerosing mesenteritis [1]. The prevalence of mesenteric panniculitis is reportedly 0.6–2.5 percent of the general population [2, 3]. The clinical presentation is variable with nonspecific systemic symptoms such as fever, chills, and generalized weakness to nonspecific gastrointestinal symptoms, such as abdominal discomfort, weight loss, constipation, or diarrhea, as well as generalized symptoms, such as fever, weakness, and chills. The exact pathophysiology is unknown, but it can be associated with cancers and autoimmune disease. Diagnosis can be usually made by CT scan, and sometimes surgical biopsy and pathological analysis are required to confirm the diagnosis.

## 2. Case Report

A 46-year-old woman presented with a 2-year history of recurrent fevers up to 38.9°C, fatigue, and frequent urination. Her symptoms were intermittent with episodes of muscle weakness, joint stiffness, and extreme headache lasting approximately 30 hours. She had no chest pain, no abdominal pain, no change in bowel habits, and no recent weight changes. Her past medical history was significant for thalassemia, dermatographism, and three early miscarriages. Her past surgical history was notable for gastric sleeve and hysterectomy complicated by pelvic abscess that required an interventional radiology drain 4 months prior to the presentation. Family history was significant for autoimmune diseases such as gout, rheumatoid arthritis, and systemic lupus erythematosus.

Her temperature was 36.6°C without any abdominal tenderness. Laboratory findings revealed a microcytic anemia with occasional schistocytes. Comprehensive metabolic panel, C-Reactive Protein (CRP), the erythrocyte sedimentation rate (ESR), HIV assay, Quantiferon tuberculosis, blood cultures, lactate dehydrogenase, anti-nuclear antibody, and serum protein electrophoresis were all within normal limits. Additional studies such as lupus anticoagulant, hepatitis virus profile, complement levels, Epstein–Barr virus acute infection, cardiolipin, rapid plasma reagin, DNA double strand, smooth muscle, Sjogren's extractable nuclear profile, beta-2-glycoprotein 1 antibodies, Smith, and ribonucleoprotein extractable nuclear were also normal.

**Figure 1:**
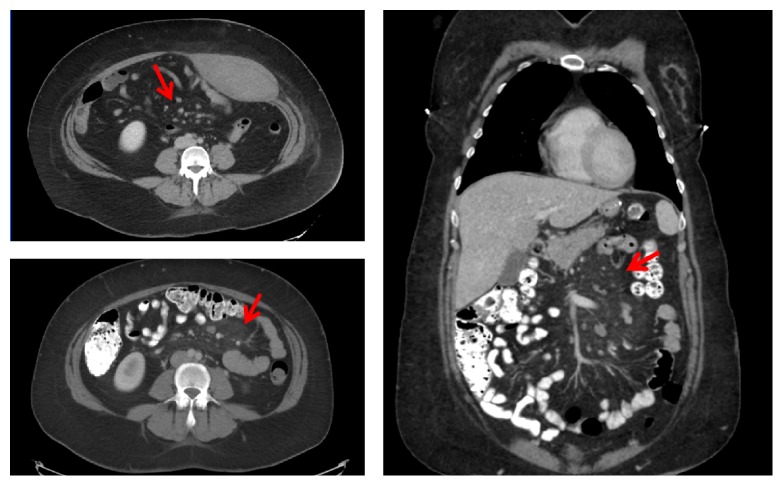
Red arrows pointing to hyperattenuated mesentery in the left hemiabdomen.

Spiral CT of the abdomen and pelvis, using a reconstructed slice thickness of 5 mm after oral and IV contrast administration, showed a misty appearance of the mesentery in the left hemiabdomen with multiple enlarged mesenteric lymph nodes measuring up to 1 cm in the short axis (Figure 1). The radiological findings likely represent chronic mesenteric panniculitis with early suggestion of it seen in previous CT abdomen 2 years a priori. The rest of the exam was significant for nonobstructing 2 mm calcification noted within the inferior pole of the left kidney.

Mesenteric biopsy revealed fibroadipose tissue with focal fat necrosis with lymphohistiocytic inflammation and fibrosis. There was no evidence of malignancy. Flow cytometry did not reveal abnormal B or T cell populations, excluding a lymphoproliferative process.

The patient was offered treatment with prednisone, but the patient was hesitant to start steroids, so methotrexate 15 mg by mouth once a week was started but had minimal response. Further treatment options are being explored.

## 3. Discussion

Mesenteric panniculitis is a rare inflammatory condition manifested by chronic and nonspecific inflammation of the adipose tissue of the intestinal mesentery. There is a 2-3 : 1 predilection for men versus women. Incidence increases with age and is extremely rare in children due to less mesenteric fat than in adults. Precise etiology remains unclear although history of abdominal surgery or trauma has been reported [4]. Histologically, the disease progresses in three stages: mesenteric lipodystrophy, mesenteric panniculitis, and retractile mesenteritis [5]. Histology in stage two reveals infiltrate of plasma cells and few polymorphonuclear leukocytes, foreign-body giant cells, and foamy macrophages. Fever, abdominal pain, and malaise are common symptoms although the disease is usually asymptomatic. Other clinical symptoms include abdominal pain, abdominal fullness, nausea, pyrexia, and weight loss [6].

Workup for mesenteric panniculitis requires a broad differential diagnosis, including evaluation for malignancy. Blood tests are usually within normal limits; however, neutrophilia, increased erythrocyte sedimentation rate, or anemia has been reported [1]. Some reports state that very few patients with this pathology can be diagnosed without surgery [7]. Therefore, workup for fever of unknown origin, which includes high-resolution CT among others, has allowed clinicians to distinguish mesenteric panniculitis from other mesenteric diseases with similar imaging features like lymphoma, desmoid tumor, and carcinomatosis. Mesenteric panniculitis presents as a heterogeneous mass with a large fat component and interposed linear bands with soft tissue density [1].

Therapy for mesenteric panniculitis is individualized on a case-by-case basis. Variety of drugs such as steroids, thalidomide, methotrexate, cyclophosphamide, progesterone, colchicine, azathioprine, tamoxifen, antibiotics, or radiotherapy has shown different degrees of success [8].

In conclusion, mesenteric panniculitis is a rare etiology of fever of unknown origin. Diagnosis of this nonspecific inflammatory disease requires a multidisciplinary approach from rheumatology, infectious disease, surgery, general internal medicine, and gastroenterology. Fortunately, diagnosis with CT scan is highly suggestive and distinctive, allowing workup for fever of unknown origin to be cost effective and efficient in making the proper diagnosis. These patients are usually frustrated by their nonspecific symptoms; therefore, patient education is paramount for a healthy patient-physician relationship. It is vital that they understand the risk factors, complications, treatment, and prognosis of their febrile illness. These patients usually have a good prognosis with low recurrence rate.
